# Enzymatic approaches to nicotinic acid synthesis: recent advances and future prospects

**DOI:** 10.3389/fbioe.2025.1585736

**Published:** 2025-06-04

**Authors:** Rahul Vikram Singh, Vipin Chandra Kalia, Krishika Sambyal, Bakul Singh, Anurag Kumar, Jung-Kul Lee

**Affiliations:** Department of Chemical Engineering, Konkuk University, Seoul, Republic of Korea

**Keywords:** biocatalysis, biotransformation, omics technology, immobilization, nitrilase

## Abstract

Biocatalyst-mediated reactions have led to revolutionary transformations in the organic synthesis of pharmaceuticals, drugs, and other chemicals. Nicotinic acid (vitamin B3) is an essential precursor for nicotinamide adenine dinucleotide (NAD^+^) biosynthesis and is vital for numerous metabolic processes. Since the human body cannot synthesize nicotinic acid, it relies on external sources. Therefore, nicotinic acid synthesis has gained huge attraction. In recent years, the industrial production of nicotinic acid has increasingly shifted from traditional chemical methods to more biocatalytic processes, leveraging the power of biocatalysts. This review highlights the biocatalyst-mediated synthesis of nicotinic-acid- and nitrile-metabolizing enzymes through state-of-the-art omics-based techniques to improve enzyme catalytic efficiency and stability via various approaches. Future research prospects and challenges associated with nicotinic acid production are also discussed.

## Graphical Abstract

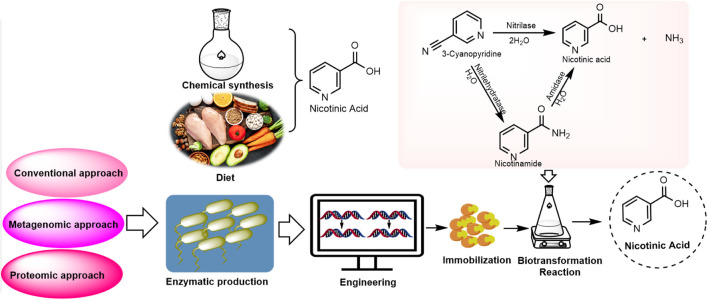

## 1 Introduction

Biocatalyst-mediated processes have provided an alternative approach to the organic synthesis of pharmaceuticals, drugs, and other chemicals, which has changed the synthesis trend. In the last 2 decades, the enzymatic process has gained huge attention in organic synthesis due to its unique properties like one-step reactions, mild reaction conditions, high selectivity, etc. (France et al., 2023). Vitamin B3 is generally known as niacin. It occurs in the form of nicotinic acid and nicotinamide and is essential to perform cellular functions. In human body, niacin is synthesized from the precursor molecule tryptophan, which is obtained from food and other sources or directly from the diet (Schmitz and Lowenstein, 2019). In late 19th century, pellagra, a disease caused by vitamin B3 deficiency, was an epidemic in southern United States, affecting more than three million people between 1906 and 1940, with more than 100,000 deaths recorded (Rajakumar 2000; Viljoen et al., 2021). In southern California, 1,396 deaths were recorded in 1915 over a period of 10 months, and approximately 100,000 people were affected in 1916 (Sugita et al., 2013; Schmitz and Lowenstein, 2019; Prabhu et al., 2021). To overcome the deficiency, niacin has been used to treat pellagra (Denu, 2005; Schmitz and Lowenstein, 2019).

Nicotinic acid has also been widely investigated for various functions, such as its effectiveness at treating patients with schizophrenia, bipolar type II disorder, and various psychiatric states (Jonsson, 2018; Noda et al., 2020; Papafaklis et al., 2024). Nicotinic acid is effective in treatment of combined hyperlipidemia and it acts as a lipid-modifying agent that significantly affects lipoproteins, with reductions in lipoprotein levels of 20%–38% (McKenney, 2004). Nicotinic acid also plays a significant role in growth and maintenance of the central nervous system. It helps regulate cholesterol levels by lowering LDL cholesterol, increasing HDL cholesterol, and maintaining triglyceride levels (Aim-High Investigators, 2011; Zio et al., 2024). Nicotinic acid is an important micronutrient, and food items such as chicken, fish, peanuts, brown rice, whole wheat, mushrooms, green peas, and potatoes serve as major nicotinic-acid-rich sources (Panda et al., 2017, https://www.healthline.com/nutrition/foods-high-in-niacin). Medications with vitamin B3 are also available to consumers, but nicotinic acid extraction from food sources is limited. Therefore, industrial production of nicotinic acid is achieved by chemical methods; however, in recent years, many alternatives have been developed because of the disadvantages of chemical production.

This review focuses on recent advances in the biocatalytic production of nicotinic acid, highlighting the biosynthesis of nicotinic acid and its production methods. It outlines two main pathways for NAD^+^ synthesis in humans: *de novo* and salvage. The article compares chemical and enzymatic methods for nicotinic acid production, highlighting the advantages of enzymatic synthesis. Various strategies to improve nicotinic acid production are explored, including nitrilase engineering and heterologous gene expression. Enzyme and genetic engineering have shown promising results in enhancing yield and efficiency. For example, mutated strains of *Acidovorax facilis* and *Pseudomonas* putida demonstrated significantly higher catalytic efficiency for nicotinic acid production. The review also covers screening approaches for new nitrilases, discussing conventional culture-dependent methods and advanced techniques like metagenomics and proteomics. While traditional methods rely on isolating and culturing microorganisms, metagenomics allows for the analysis of entire microbial communities without the need for cultivation. Overall, the article emphasizes the potential of enzymatic processes and genetic engineering in enhancing nicotinic acid production for industrial applications. It highlights the ongoing research efforts to improve yield, efficiency, and sustainability in nicotinic acid synthesis.

## 2 Biosynthesis pathways of nicotinic acid

**FIGURE 1:**
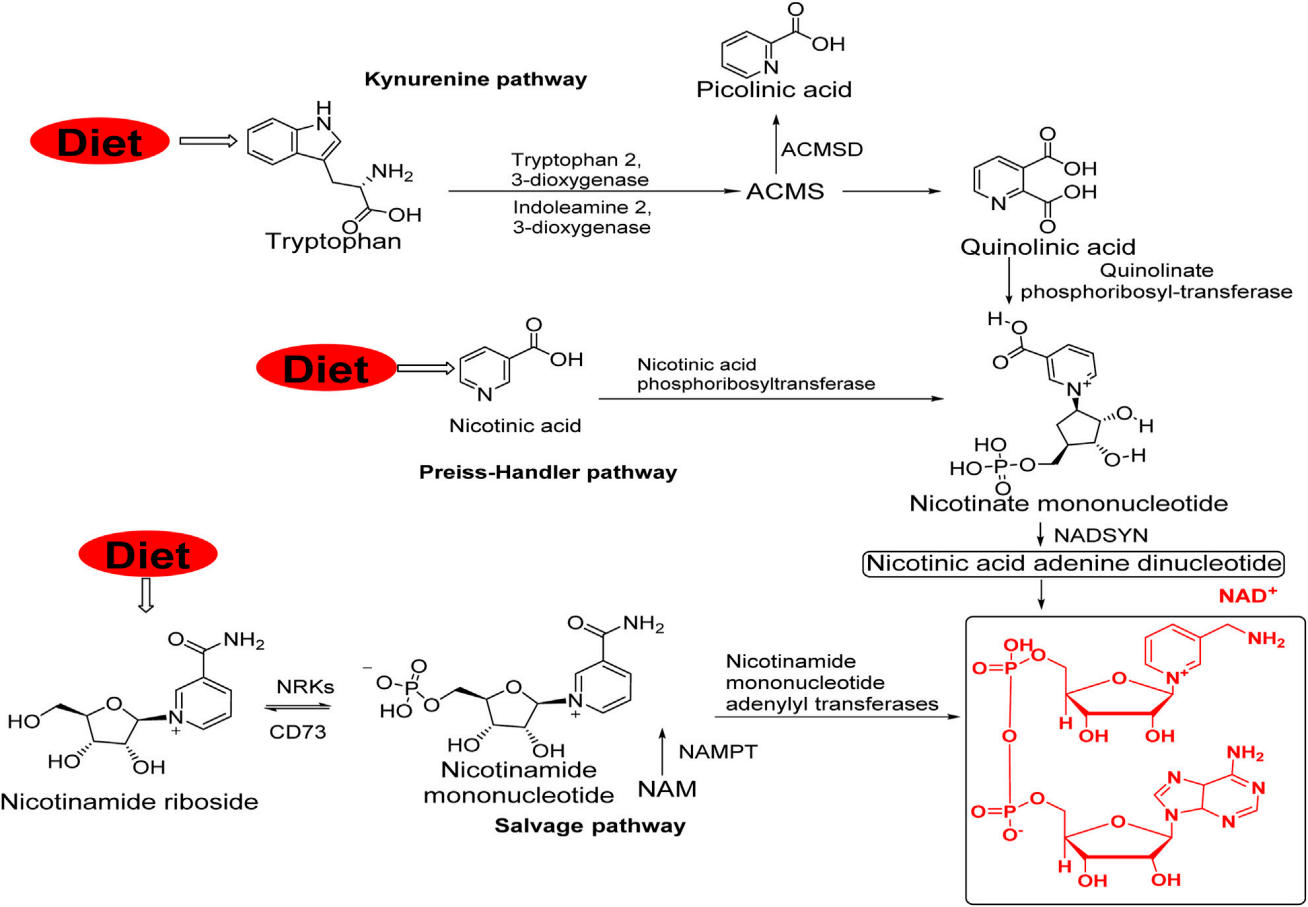
Schematic representation of NAD^+^ synthesis pathways, *de novo* from tryptophan via the kynurenine pathway or from nicotinic acid via the Preiss–Handler pathway and the salvage pathway from nicotinamide (NAM) (Xie et al., 2020). Abbreviations: IDO, indoleamine 2,3-dioxygenase; QA, quinolinic acid; NAMN, nicotinate mononucleotide; QPRT, quinolinate phosphoribosyl-transferase; NAPRT, nicotinic acid phosphoribosyltransferase; NMNATs, nicotinamide mononucleotide adenylyl transferases; NADSYN, NAD synthase; NR, nicotinamide riboside; Trp, tryptophan; NADKs, NAD^+^ kinases; PARPs, poly (ADP-ribose) polymerases; NNT, nicotinamide nucleotide transhydrogenase; TDO, tryptophan 2,3-dioxygenase; SARM1, sterile alpha and TIR motif-containing 1; NNMT, Nicotinamide N-methyltransferase; NMN, nicotinamide mononucleotide; PUFAs, polyunsaturated fatty acids; NAM, nicotinamide; ACMSD, alpha-amino-beta-carboxy-muconate-semialdehyde decarboxylase).

The human body cannot directly synthesize nicotinic acid. Therefore, humans depend on dietary intake to meet their nicotinic acid requirements. Nicotinic acid is a precursor for biosynthesis of nicotinamide adenine dinucleotide (NAD^+^), which is an essential co-factor for cellular regulation to support various metabolic functions (Belenky et al., 2007; Imai and Guarente, 2014; Cantó et al., 2015; Garten et al., 2015). In human body, NAD^+^ is synthesized through two pathways: *de novo* and salvage pathways (Figure 1) (McReynolds et al., 2020).

### 2.1 De novo pathway

In mammalian cells, tryptophan 2,3-dioxygenase or indoleamine 2,3-dioxygenas catalyze kynurenine pathway to obtain NAD^+^ from dietary tryptophan (Figure 1). As an intermediate, α-amino-β-carboxy-muconate-semialdehyde can be cyclized to quinolinic acid, while α-amino-β-carboxy-muconate-semialdehyde decarboxylase converts α-amino-β-carboxy-muconate-semialdehyde to picolinic acid, which limits the flux from tryptophan to NAD^+^ (Katsyuba et al., 2018). An important step in the pathway to NAD^+^ biosynthesis is the conversion of quinolinic acid to nicotinate mononucleotide by quinolinate phosphoribosyltransferase (Youn et al., 2016; Badawy, 2017). Dietary nicotinic acid can be converted to nicotinate mononucleotide by nicotinic acid phosphoribosyltransferase through the Preiss-Handler pathway (Marletta et al., 2015. Nicotinamide mononucleotide adenylyl transferases catalyze the production of nicotinamide adenine dinucleotides (NAADs). NAADs are later converted to NAD^+^ by NAD synthase using glutamine as a nitrogen donor (Brazill et al., 2017; Xie et al., 2020).

### 2.2 Salvage pathway

To maintain cellular NAD^+^ levels, most NAD^+^ is recycled from its precursors, such as nicotinamide mononucleotide, nicotinamide, nicotinic acid, and nicotinamide riboside, in the salvage pathway (Figure 1) (Braidy et al., 2019). Among these, nicotinamide mononucleotides can be recycled from NAD^+^ consumption reactions via both NAD^+^ -dependent deacylation and ADP ribosylation into nicotinamide mononucleotides by nicotinamide phosphoribosyltransferase. This catalyzes the rate-limiting reaction in the salvage pathway (Wang et al., 2006). Equilibrative nucleoside transporters import the precursor nicotinamide riboside to transform it into a nicotinamide mononucleotide using nicotinamide riboside kinases (NRK1/2) (Rajman et al., 2018). Finally, nicotinamide mononucleotide adenylyl transferases adenylate nicotinamide mononucleotide to yield NAD^+^ (Xie et al., 2020; Zhou et al., 2002; Werner et al., 2002).

## 3 Nicotinic acid production

### 3.1 Chemical synthesis

**FIGURE 2:**
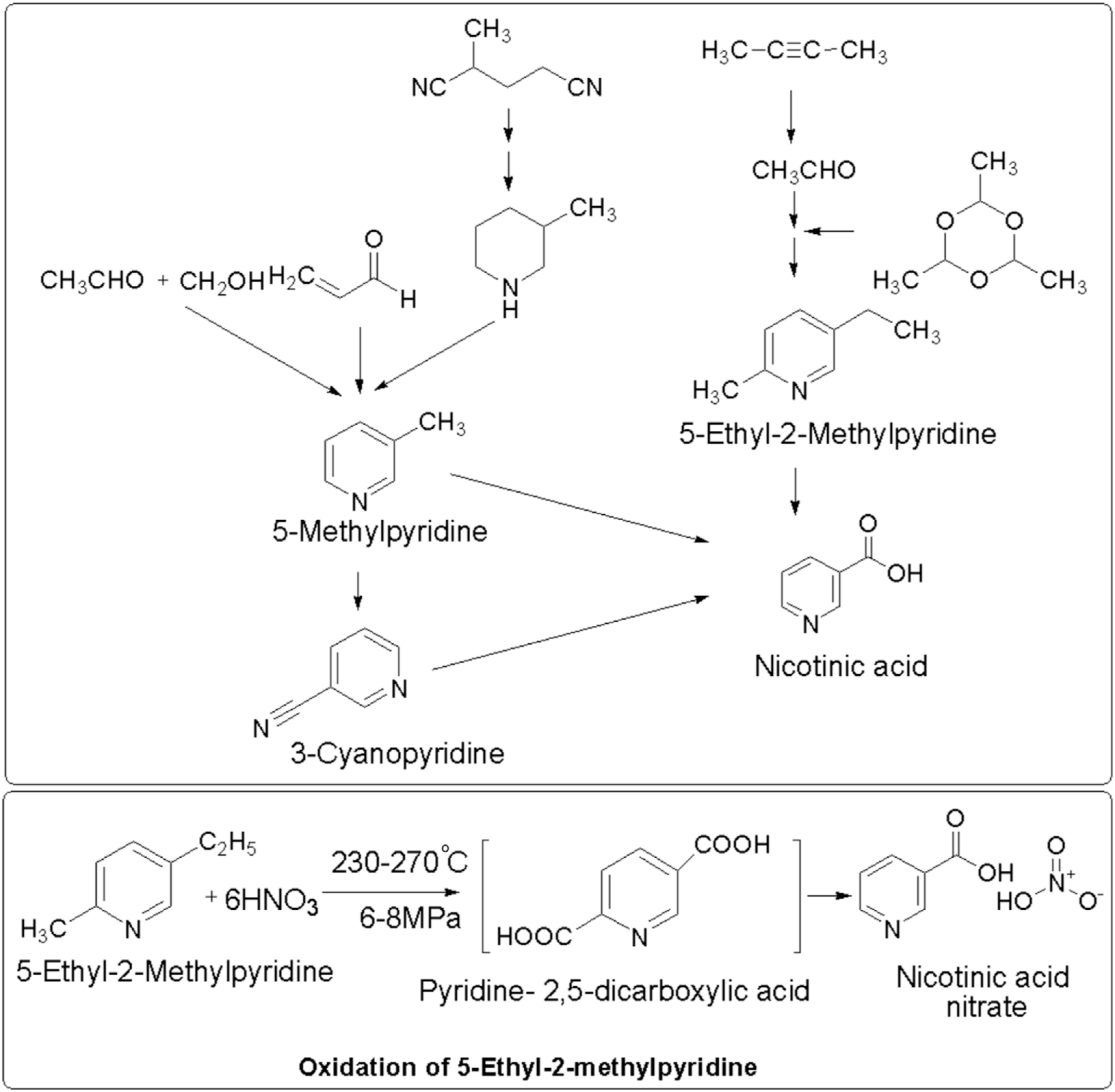
Chemical synthesis of nicotinic acid.

Conventionally, nicotinic acid was produced using chemical methods, such as ammoxidation or liquid-phase oxidation (Figure 2), which require harsh production conditions (>150°C); a high cost to fulfil the requirement of metal catalysts; expensive equipment; and unwanted waste generation including by-products and inorganic salts (hydrogen cyanide, sodium chloride, etc.). However, this method provides modest yields (generally 80%–90%) (Chuck, 2005; Raja et al., 2008; Jin et al., 2013; Lisicki et al., 2022). The chemical synthesis of nicotinic acid offers a high yield using a well-established process with commercially available materials. However, it generates toxic NOx by-products; requires high pressure and temperature; uses excess corrosive nitric acid; has a low atom economy (25%); and is environmentally unfriendly, producing more than 1 t of CO2 per t of niacin (Lisicki et al., 2022).

### 3.2 Enzymatic synthesis

**FIGURE 3:**
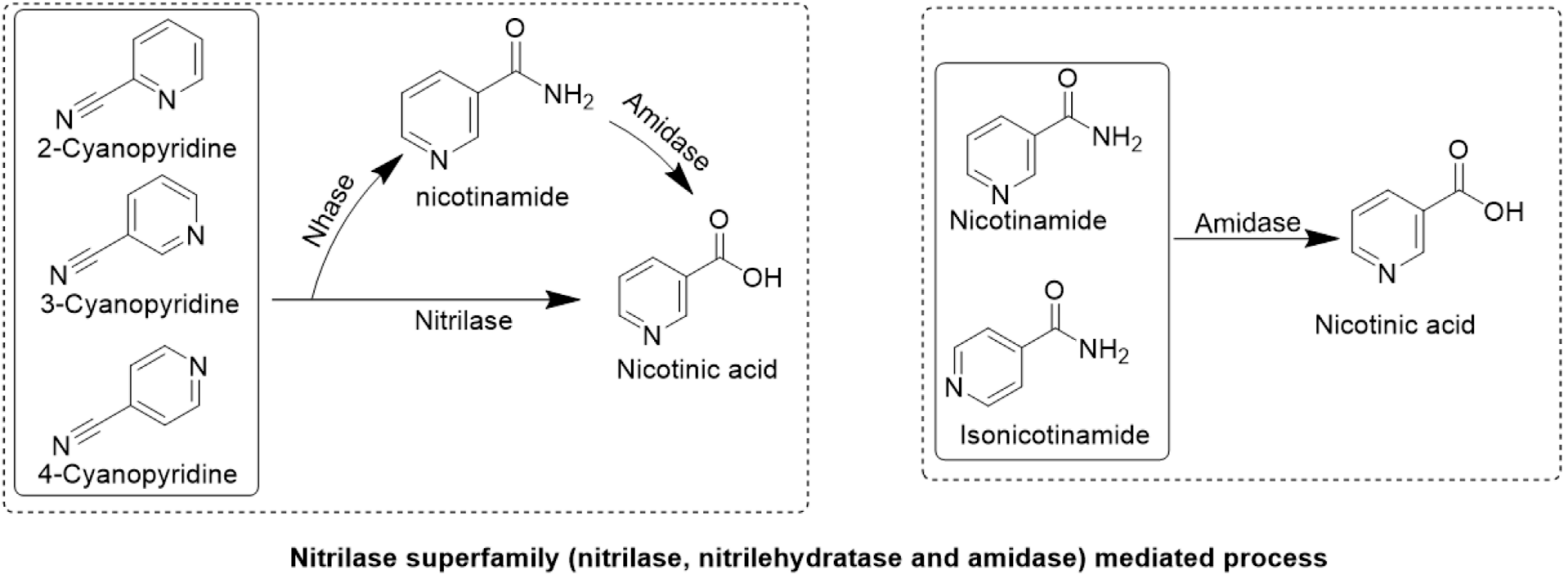
Enzymatic synthesis. Conversion of nitrile substrates (2-cyanopyridine, 3-cyanopyridine, 4-cyanopyridine) to nicotinic acid by nitrilase or conversion of amide substrates (nicotinamide, isonicotinamide) to nicotinic acid by amidase. Nitrilehydratase (Nhase) converts nitriles to nicotinamide.

In contrast to chemical methods, the biocatalyst-mediated eco-friendly synthesis of nicotinic acid is a significant method in synthetic organic chemistry that has gained considerable attention in recent years because of its high conversion rates under mild reaction conditions (Table 1) (Gong et al., 2016; Gong et al., 2018; Gong et al., 2012). The conventional process of nicotinic acid production involves high energy consumption because of its high reaction temperature (Fan et al., 2017). Therefore, industries are adopting enzymatic processes to produce nicotinic acid. In enzymatic synthesis, microbial hydrolytic enzymes are the main producers of nicotinic acid through the conversion of commercially available substrates, such as 2-cyanopyridine, 3-cyanopyridine, 4-cyanopyridine, nicotinamide, and isonicotinamide, in a single-step bioconversion (Chuck, 2005). Mathew et al. (1988). Reported the first nitrilase-catalyzed process for the production of nicotinic acid from 3-cyanopyridine by *Rhodococcus rhodochrous* J1, with a 100% yield (Mathew et al., 1988; Zheng et al., 2018). Subsequently, various nitrilases (Table 2) were investigated for their ability to produce nicotinic acid using different substrates (Figure 3) (Salman et al., 2022). To date, various nitrilases, including *Bacillus* pallidus Dac521 (Almatawah and Cowan, 1999), Rhodococcus sp. NDB 1165 (Prasad et al., 2007), *Nocardia* *globerula* NHB-2 (Sharma et al., 2006; Malandra et al., 2009), Fusarium proliferatum ZJB-09150 (Jin et al., 2013), have been reported to produce nicotinic acid via whole-cell catalysis of 3-cyanopyridine hydrolysis. On the bench scale, wild-type strains perform well; however, problems arise at the industrial level. Most wild-type strains do not have the potential to produce large-scale products for industrial production. Therefore, enzyme and genetic engineering of such strains can help improve yield. Previously reported nitrilases have several limitations, including a low substrate tolerance and long bioconversion time for the production of nicotinic acid.

**TABLE 1:** Methods reported for nicotinic acid production—advantages and disadvantages.

Method	Advantages	Disadvantages	References
Chemical	High efficiency and yield: The industrial oxidation of 5-ethyl-2-methylpyridine achieves 96% conversion and 91% yield Commercially available raw materials: Available, e.g., 5-ethyl-2-methylpyridine Scalability: Chemical synthesis methods are suitable for large-scale industrial production to meet market demands Versatility: Different chemical approaches exist, such as oxidation of 5-ethyl-2-methylpyridine and ammoxidation of 3-picoline, allowing for flexibility in production methods Direct routes: Some methods, such as the direct oxidation of 3-picoline, offer more straightforward pathways to nicotinic acid	Environmental concerns: Most established chemical synthetic methods use non-renewable materials derived from petroleum as raw materials Waste management: Chemical synthesis often results in large quantities of toxic wastes, including spent catalysts, requiring thorough management and significant expenses for disposal Energy requirements: Requires high temperature and pressure, leading to high energy consumption Corrosive conditions: Some methods, such as the oxidation of 5-ethyl-2-methylpyridine, involve highly corrosive reaction environment By-products: Processes such as the oxidation of 5-ethyl-2-methylpyridine produce harmful by-products, such as NOx Dependence on petroleum: The use of petroleum-derived raw materials makes these processes susceptible to fluctuations in petroleum prices and availability	Chuck 2005 Raja et al. (2008) Jin et al. (2013) Lisicki et al. (2022)
Enzymatic	Milder reaction conditions: Requires mild conditions Higher selectivity: Enzymes are highly specific, reducing unwanted side reactions and improving product purity Environmentally friendly: Biological processes generally produce less toxic waste and use renewable resources Potential for continuous production: Immobilized enzyme systems allow for semi-continuous or continuous production Versatility: Different microbial strains can be engineered to optimize production pathways	Low yields Feedback inhibition: NAD can inhibit enzymes such as aspartate oxidase and NAD synthetase Enzyme purification: Sometimes the purified form of the enzyme is required for the process Limited substrate tolerance Slower conversion rates	Prasad et al. (2007) Gong et al. (2017) Gong et al. (2018) Dai et al. (2022) Monika et al., 2023 Liu et al. (2023)
Others	Easily available in plants and animals	Low extraction yield and availability of resources. Requires advanced purification setup	Chen et al. (2008) Çatak (2019) Zhang et al. (2021)

**TABLE 2:** Wild-type strains reported for the production of nicotinic acid.

Microbes	Substrate/concentration	Nicotinic acid production conditions	Mode of reaction	Nicotinic acid yield	Reference
*Rhodococcus rhodochrous J1*	200 mM 3-cyanopyridine	Temp: 25°C, pH: 8.0 Time: 26 h	Fed Batch	100%	Mathew et al. (1988)
*Bacillus* pallidus Dac521	100 mM 3-cyanopyridine	Temp: 50°C–60°C, pH: 8.0 Time: 100 h	Fluidized bed bioreactor	100%	Almatawah and Cowan (1999)
*Nocardia* globerula NHB-2	40 mM 3-cyanopyridine	Temp: 25°C, pH: 7.5 Time: 9 h	Fed Batch	98.6%	Sharma et al. (2006)
*Rhodococcus* sp. NDB 1165	1.6 M 3-cyanopyridine	Temp: 40°C, pH: 8.0 Time: 26 h	Fed Batch	100%	Prasad et al. (2007)
*Aspergillus niger* K10 *F. solani*	50 mM 4- cyanopyridine	Temp: 35°C–40°C, pH: 8.0 Time: 2–38 h	Continuous stirred membrane reactor	>90% 98%	Malandra et al. (2009)
*Nocardia* globerula NHB-2	300 mM 3-cyanopyridine	Temp: 25°C, pH: 8.0 Time: 200 min	Fed Batch	57%	Sharma et al. (2011)
*Rhodobacter sphaeroides* LHS-305	200 mmol L−1 3-cyanopyridine	Temp: 30°C, pH: 9.0 Time: 13 h	Batch	93%	Yang et al. (2011)
*Nocardia* globerula NHB-2	700 mM 4-cyanopyridine	Temp: 35°C, pH: 8.0 Time: 140 min	Fed batch	100%	Sharma et al. (2012)
*Fusarium proliferatum* ZJB-09150	60 mM 3-cyanopyridine	Temp: 50°C–55°C, pH: 9.0 Time: 15 min	Shake-flask	98.9%	Jin et al. (2013)
*Rhodococcus pyridinivorans* SN2	10.5 g of 3-cyanopyridine	Temp: 50°C–55°C, pH: 9.0 Time: 6.5 h	Biphasic fed batch	100%	Kameswaran et al. (2014)
*Geobacillus subterraneus* RL-2a	0.72 M nicotinamide	Temp: 70°C, pH: 6.5 Time: 400 min	Fed batch	100%	Mehta et al. (2014)
*Stenotrophomonas maltophilia* AC21	>70 mM 3-cyanopyridine	Temp: 45°C, pH: 8.0 Time: 6 h	Fed batch	90%	Badoei-Dalfard et al. (2016)

## 4 Strategies to further improve nicotinic acid production

### 4.1 Nitrilase engineering for nicotinic acid production

Many attempts have been made to produce nicotinic acid using enzyme/genetic engineering. In addition to extensive research on recombinant strains and genes associated with nitrilase, synthetic biology has been used to construct microorganisms that overproduce nitrilase for the production of nicotinic acid (Table 2). The enzyme/genetic engineering of some microbes enhances the bioconversion rate of 3-cyanopyridine/nicotinamide to nicotinic acid. Wild-type strains have several limitations; however, nitrilase-mediated processes also have some restrictions, such as low catalytic efficiencies. To overcome this limitation, Li et al. (2016) improved the catalytic efficiency of NitA from Acidovorax facilis 72 W for nicotinic acid production through site-directed mutagenesis. The mutated NitA-C2 (F168V-S192F) showed a five-fold increase in specific activity towards 3-cyanopyridine. Both nitrilases had an optimal pH in the range of 6.0–8.0 and an optimal temperature of 60°C, but NitA-C2 had decreased stability. Whole-cell catalysis achieved 100% conversion of 0.1 mol L−1 3-cyanopyridine. *E. coli* expressing NitA-C2 demonstrated a three-fold higher conversion rate (1.0 mmol min−1 g−1 wet cell weight) compared to the original strain. These results suggest that mutated NitA-C2 is a promising candidate for industrial-scale biological nicotinic acid production (Li et al., 2016). Gong et al. (2017) performed site-saturation mutagenesis of different amino acids (Asn40, Phe50, and Gln207) in a recombinant nitrilase from *Pseudomonas* putida CGMCC3830. The mutants N40G, F50W, and Q207E showed a two-fold higher yield of nicotinic acid than the wild-type strain. Double and triple mutations were introduced, and four mutants (N40G/F50W, N40G/Q207E, F50W/Q207E, and N40G/F50W/Q207E) were generated to increase the catalytic efficiency. The triple-mutant N40G/F50W/Q207E showed 87% higher catalytic efficiency than the wilt-type strain towards 3-cyanopyridine for nicotinic acid production (Gong et al., 2017; Jin et al., 2024) conducted a study to improve the nitrilase AfNIT for the enzymatic hydrolysis of terephthalonitrile to 4-cyanobenzoic acid. Virtual screening identified key mutation sites, resulting in a triple-mutant with 3.8 times higher activity than the wild-type strain. The mutant achieved 98.7% conversion of 150 g/L terephthalonitrile, demonstrating potential for industrial biomanufacturing of 4-cyanobenzoic acid (Jin et al., 2024). The specific activity of the NitA gene from *Acidovorax facilis* 72 W increased by five-fold to 35 U/mg of protein after introducing the mutation (Li et al., 2016). Yield improvements through engineering have proven that mutagenesis has huge potential for future studies in which the yield of nicotinic acid can be improved by several-fold at a higher scale. Monika et al. (2023) conducted a study to improve the nitrilase efficiency of Gordonia terrae by converting 3-cyanopyridine to nicotinic acid through chemical mutagenesis. The N-methyl-N-nitro-N-nitrosoguanidine generated mutant MN12 showed a significant increase in nitrilase activity. Optimization of the culture conditions further enhanced enzyme production. Whole-cell catalysis achieved 100% conversion of 100 mM 3-cyanopyridine in 15 min under optimized conditions. The mutant MN12 exhibited a higher product formation rate and volumetric productivity than wild-type *G. terrae.* The recovered product was confirmed to have high purity (>99.9%) using various analytical methods. These results suggest that the mutant MN12 of *G. terrae* is a promising biocatalyst for large-scale nicotinic acid synthesis (Monika et al., 2023).

### 4.2 Heterologous gene expression for nicotinic acid production

Fan et al. (2017) cloned and overexpressed a novel nitrilase gene (REH16) from *Ralstonia eutropha* H16 in *E. coli* BL21 (DE3). The recombinant strain completely hydrolyzed 100 mM 3-cyanopyridine to nicotinic acid. In the fed-batch reaction mode, 1,050 mM 3-cyanopyridine was completely converted into nicotinic acid in 20.8 h (Fan et al., 2017). Gong et al. (2018) cloned and expressed the nitrilase gene from *P. putida* in *E. coli* BL21 (DE3) (pET-3b-NIT) and generated high-density cultures for nicotinic acid production. Furthermore, the recombinant strain was used to catalyze 200 mM 3-cyanopyridine in fed-batch mode. After 290 min of incubation, 541 g·L-1 of nicotinic acid accumulated through 22 batches. This is the highest nicotinic acid produced by recombinant nitrilase in fed-batch mode (Gong et al., 2018). Zhu et al. (2013) cloned and expressed a nitrilase gene from *Pseudomonas* putida CGMCC3830 in *E. coli* through consensus-degenerate hybrid oligonucleotide primer polymerase chain reaction (PCR), degenerate PCR, and thermal asymmetric interlaced PCR. After sequence analysis, it was observed that the open reading frame comprised 1,113 bp encoding a protein of 370 amino acids. These sequences showed a similarity of 61.6% with nitrilase from *Rhodococcus rhodochrous* J1. The *Km* and *Vmax* values for 3-cyanopyridine were determined to be 27.9 mM and 84.0 U/mg, respectively (Zhu et al., 2013).

## 5 Screening approaches for new nitrilases for nicotinic acid production

The screening of new nitrilases for nicotinic acid production can be summarized in two techniques: conventional culture-dependent approaches and advanced techniques, such as metagenomics and proteomics. Overall, metagenomic and proteomic approaches offer more comprehensive and functionally relevant insights into microbial communities than traditional culture methods, although they have their own technical and analytical challenges. The efficiency of the proteomic approach has gained considerable attention for identifying various industrially important enzymes, stimulating its use in the discovery of novel nitrilase-metabolizing enzymes (Brenner, 2002; Pace and Brenner, 2001). Functional proteomics has been used to gain structural insights into the molecular recognition of nitrilase-1 (Nit1) and nitrilase-2 (Nit2) (Barglow et al., 2008).

### 5.1 Conventional culture-dependent approach

The conventional culture-dependent approach includes the isolation of natural nitrilase/amidase-producing microorganisms that hydrolyse 3-cyanopyridine/nicotinamide into nicotinic acid and optimization of the conditions for nicotinic acid synthesis (Table 1). In this approach, microorganisms exhibiting the desired enzyme are enriched by the addition of a substrate and under suitable cultivation conditions. The selected strains are further taxonomically classified, and molecular characterization of the enzyme is performed. The nitrilase-catalyzed conversion of 3-cyanopyridine into nicotinic acid has low substrate tolerance, which creates a problem for higher production at the pilot scale, resulting in enzyme imbibition, thereby decreasing the rate of bioconversion. Therefore, the application of fed-batch reactions under such conditions may be beneficial. The strain *Nocardia* globerula NHB-2 was utilized for the production of nicotinic acid from 3-cyanopyridine (100 mM) using a fed-batch reaction at a 40 mL scale with 20 feedings. After completion of the reaction, 1,136 mM nicotinic acid was obtained. Upon process scale-up to a 1 L scale (100 mM 3-cyanopyridine) with 10 feedings (0.1 mol in 20 min) in 200 min, the rate of nicotinic acid formation reached 24.6 g h-1 g-1 dry cell weight (DCW) (Sharma et al., 2011). Similarly, S. maltophilia AC21 strain was utilized for nicotinic acid production in a fed-batch mode. After six feeding of 3-cyanopyridine (>70 mM), 96% (565 mM) nicotinic acid was produced without any enzyme inhibition. Furthermore, a fed batch was scaled-up to 1 L scale (420 mM of 3-cyanopyridine fed in six feedings of 70 mM at 40 min), and after 10 h of incubation, 90% of 3-cyanopyridine was converted to nicotinic acid (Badoei-Dalfard et al., 2016). The fed-batch reaction (500 mL, 100 mM 3-cyanopyridine feed) of strain Ralstonia eutropha H16 was performed in two stages. After 13 feedings of 1,050 mM 3-cyanopyridine in 20.8 h of incubation, 129.2 g/L of nicotinic acid was obtained (Fan et al., 2017). Recombinant nitrilase from *E. coli* BL21 (DE3) (pET-3b-NIT) was used in a fed-batch reaction (200 mM 3-cyanopyridine). After 17 feedings in 410 min, conversion reached up to 22.90 g·h-1 (Gong et al., 2018). The conventional approach relies on screening and culturing microbes in a controlled laboratory environment, using enrichment cultivation to grow the microbes under specific conditions to isolate functional organisms. This method allows researchers to target and study microbes with specific roles, such as pollutant degradation. However, it has a significant limitation, as many microbes cannot be cultured under laboratory conditions, leading to an incomplete understanding of microbial diversity (Kapinusova et al., 2023; Garg et al., 2024).

### 5.2 Metagenomics

**FIGURE 4:**
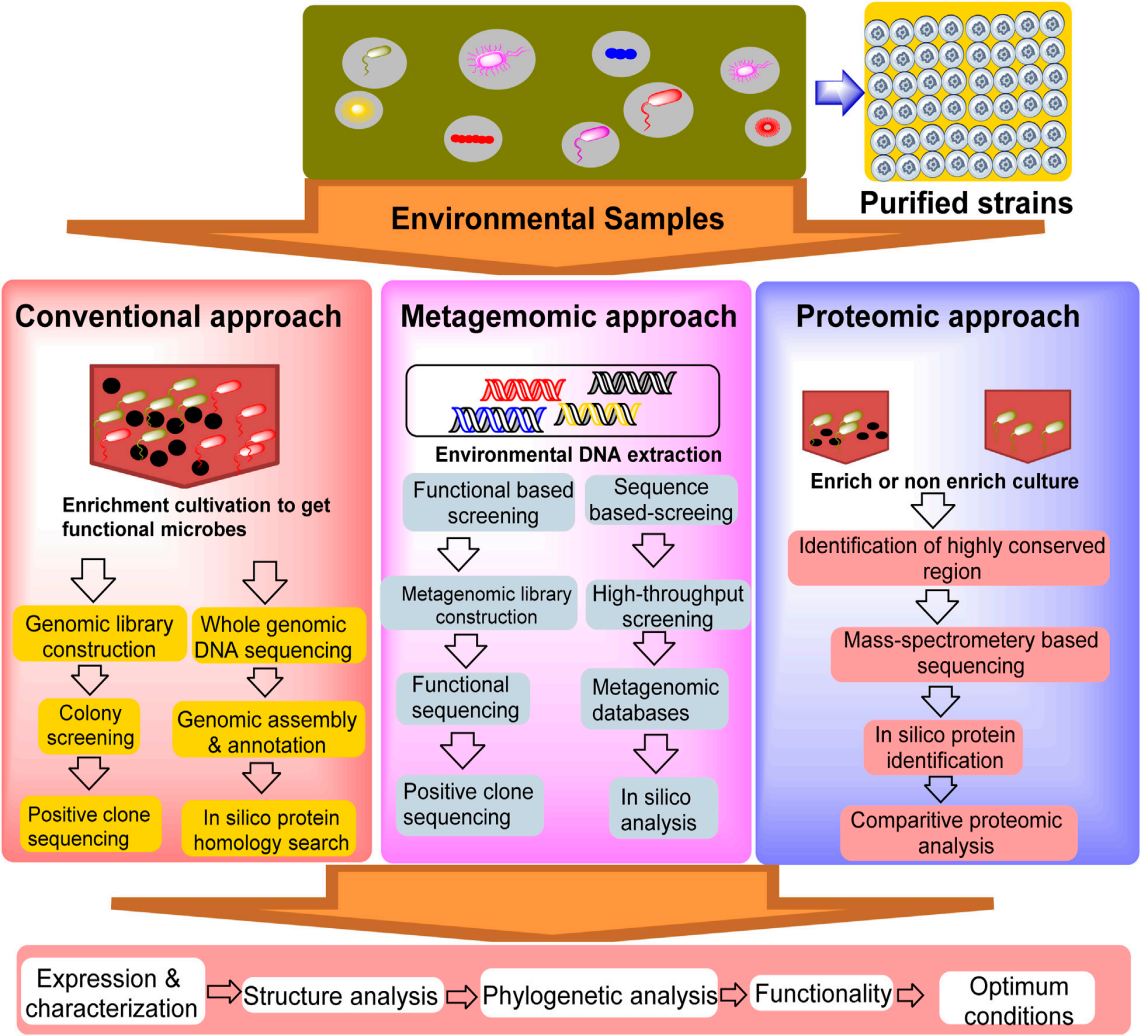
Schematic illustration of the conventional and proposed omics-based approaches for the discovery of enzymes utilized in the synthesis of nicotinic acid (adopted and modified from Barglow et al., 2008; Zhu et al., 2022).

Advanced techniques, such as metagenomics and proteomics, may be reliable for increasing the possible hit rate in the mining of nitrile-metabolizing enzymes. The flowchart presented in Figure 4 describes the various approaches used to mine nitrilase-metabolizing enzymes from the environment. The metagenomic approach bypasses the need for culture by directly extracting and sequencing DNA from environmental samples. This approach identifies genetic regions that are conserved across species, enabling the detection of a wide range of organisms, including non-culturable organisms. During the expression of uncultured microbes, protein expression faced difficulties; therefore, addressing the complexity of protein expression patterns requires a multi-faceted approach, integrating enzyme and genetic engineering with advanced screening techniques. Enzyme engineering, exemplified by site-directed mutagenesis, can enhance catalytic efficiency, as demonstrated by the five-fold increase in activity of *Acidovorax facilis* NitA mutants (Li et al., 2016). Heterologous gene expression, such as cloning nitrilases from Ralstonia eutropha in *E. coli*, offers controlled production environments (Fan et al., 2017). High-throughput sequencing technologies allow for the rapid and comprehensive analysis of microbial genomes, providing a more accurate representation of microbial diversity. The major advantage of metagenomics is its ability to capture the entire microbial community, although the sheer volume of data can present challenges for analysis and interpretation (Nwachukwu and Babalola, 2022). Furthermore, optimizing culture conditions and employing fed-batch reactions refine protein production, enhancing yields and bioconversion rates. These strategies, when combined, offer a robust framework for improving nicotinic acid production by enhancing yields, efficiency, and sustainability. Riffiani et al. (2017) screened nitrilase genes in contaminated soil from the Lombok gold mine using a metagenomic approach. DNA was extracted from the soil samples and amplified using the H1F-H1R primers. BLASTN analysis revealed high homology between the amplified fragment and the nitrilase gene of Rhodococcus rhodochrous strain tg1-A6, confirming the presence of nitrilase genes in the soil sample (Riffiani et al., 2017). Soares Bragança et al. (2017) used a metagenomic approach to discover novel nitrile-hydrolyzing enzymes from soil samples in Ireland. Nitrile compounds are versatile intermediates that are important in pharmaceutical and chemical industries. A fosmid DNA library was created from metagenomic DNA and screened in *E. coli* for enzyme activity using β-hydroxynitriles as substrates. This resulted in the identification of 33 active clones. Gene screening for nitrilase, nitrile hydratase, and amidase was performed using PCR, resulting in partial gene sequences. Ongoing studies aim to determine the complete sequences for cloning and expression, with the goal of realizing the commercial potential of these enzymes for various industrial applications (Soares Bragança et al., 2017). Sunder et al. (2020) identified nine bacterial nitrilases using genome mining and evaluated their activities on 23 industrial nitrile substrates. Nitrilases from *Zobellia galactanivorans*, *Achromobacter insolitus*, and *Cupriavidus necator* have demonstrated high activity and have been used as whole-cell biocatalysts in lab-scale processes. These enzymes efficiently convert various nitriles, including 4-cyanopyridine, iminodiacetonitrile, and mandelonitrile, with high yields and with rapid reaction times. Z. galactanivorans nitrilase produced 1.79 M isonicotinic acid in 3 h, A. insolitus nitrilase achieved 86% conversion of iminodiacetonitrile in 1 h, and C. necator nitrilase performed enantioselective hydrolysis of mandelonitrile in 4 h. These results suggest significant potential for these nitrilases in large-scale industrial biocatalytic applications for the green synthesis of pharmaceutical precursors and fine chemicals (Sunder et al., 2020). Achudhan et al. (2023) conducted ametagenomic study aimed at discovering novel nitrilases from coal metagenomes using *in silico* mining to address the environmental and health risks posed by toxic nitriles. The coal metagenomic DNA was sequenced, assembled, and annotated. The nitrilase sequences were identified and analyzed for phylogeny, conserved regions, and physicochemical properties. The 2D and 3D structures were predicted using various tools, including AlphaFold2. A novel nitrilase from unclassified Alphaproteobacteria was identified, and its 3D structure was predicted with high confidence (95.8%) and was verified through molecular dynamics simulation. Molecular docking analysis revealed binding affinities similar to other prokaryotic nitrilases, with minimal deviation (±0.5), suggesting potential applications in nitrile degradation (Achudhan et al., 2023). Metagenome mining enables targeted exploration of unique genetic sequences to identify novel biocatalysts without multiple rounds of evolution. This approach complements the traditional methods for discovering sustainable catalysts. This provides researchers with optimized starting points for the evolution of promiscuous biocatalysts and increases our understanding of enzyme classes, including conserved residues. This knowledge facilitates the faster evolution of specialized enzymes. Metagenomics is expected to remain a powerful tool in biocatalytic research, offering efficient pathways for developing enzymes for various applications in sustainable chemistry (Hogg et al., 2024).

### 5.3 Proteomics

Proteomics is an advanced approach that can play an important role in the discovery of novel enzymes. It directly detects and quantifies protein expression from an extensive repository of microbial sources for biotechnological applications (Bers et al., 2011; Sturmberger et al., 2016). The proteomic approach (Figure 4) focuses on studying proteins produced by microbial communities to understand their functional roles. It uses *in silico* tools for protein identification, and mass spectrometry for direct protein analysis, thereby enabling comparative studies of protein expression under different environmental conditions. Proteomics links genetic data with actual biological activities and offers insights into the structure, function, and evolutionary relationships of microbial proteins (Birhanu, 2023). Typically, a highly conserved cysteine nucleophile, a glutamate base, is the main target when screening for potential nitrilase-metabolizing enzymes, because all nitrilases exhibit a common catalytic mechanism that contains a highly conserved cysteine nucleophile, glutamate base, and conserved active-site lysine that completes the catalytic triad (Zhu et al., 2022). Although this approach provides detailed functional information, it is limited by complexity of the protein expression patterns and need for advanced analytical tools. Choi et al. (2016) conducted proteomic and functional analyses, which revealed that Arabidopsis nitrilases are crucial for plant defense against *Pseudomonas syringae* pv. Tomato (Pst). AtNIT2, AtNIT3, and AtNIT4 are induced by Pst infection, with AtNIT2 and AtNIT4 showing significant induction by avirulent Pst DC3000 (avrRpt2). Transgenic and mutant nitrilase lines exhibit increased susceptibility to Pst and *Hyaloperonospora arabidopsidis*. NIT2 overexpression leads to higher Pst growth in the leaves. The nit2 mutation enhances Pst growth in salicylic acid (SA)-deficient plants. Arabidopsis nitrilase 2 is involved in indole-3-acetic acid signaling for defense and R-gene-mediated resistance. It regulates SA-mediated resistance to avirulent Pst, but is not required for defense against virulent Pst (Choi et al., 2016). As summarized in Table 3, many genes encoding nitrilases transform nitrile/amide substrates into nicotinic acid. The exploitation of *E. coli* has rapidly increased in omics technologies for nitrilase-producing strains because it is a selective and efficient approach to identifying nitrilase-metabolizing genes and their expression in *E. coli* as a host. In recent years, omics has gained significant attention for nitrilase production. Several recombinant nitrilases have been successfully cloned and characterized to produce nicotinic acid with improved specific activity by several-fold (Gong et al., 2017). *Pseudomonas* *putida* CGMCC3830 showed 84.0 U/mg specific activity towards 27.9 mM 3-cyanopyridine (Zhu et al., 2013).

**TABLE 3:** Recombinant strains reported for the production of nicotinic acid.

Native microbes	Recombinant host	Enzyme	Substrate and concentration	Nicotinic acid production conditions	Mode of reaction	Nicotinic Acid yield	Reference
*Pseudomonas* *putida* X3 mutant	-	Nitrilase	4.6 mol/L 4-cyanopyridine	Temp: 40°C, pH: NA Time: 630 min	Batch	100% of isonicotinic acid	Qian et al. (2021)
*Pantoea* sp. (Pa-Ami)	*E. coli* BL21(DE3)	Amidase	370 mM chlorinated nicotinamides	Temp: 40°C, pH: 8.0 Time: 450 min	Fed-batch	94.2%	Pai et al. (2014)
*Pseudomonas putida*	*E. coli* BL21 (DE3) (pET-3b-NIT)	Nitrilase	200 mM 3-cyanopyridine	Temp: 30°C, pH: 7.2 Time: 290 min	Fed batch	99.87%	Gong et al. (2018)
*Ralstonia eutropha* H16	*E. coli* BL21(DE3)	Nitrilase	1,050 mM 3-cyanopyridine	Temp: 37°C, pH: 6.6 Time: 20.8h	Fed batch	99.95%	Fan et al. (2017)
*Acidovorax facilis* 72W ATCC 55746	*E. coli* BL21 (DE3-pET-nitA-C2)	Nitrilase	0.1 mol L-1 3-cyanopyridine	Temp: 37°C, pH: 7.0 Time: 10 min	Batch	100%	Li et al. (2016)
*A. faecalis* MTCC 126	*E. coli* JM109	Nitrilase	1 M 3-cyanopyridine	Temp: 37°C, pH: 7.5 Time: 5 h	Batch	93%	Pai et al. (2014)
*Pseudomonas* *putida* CGMCC3830	*E. coli* strains JM109 & *Rosetta-gami* (DE3)	Nitrilase	10–150 mM cyanopyridine	Temp: 40°C, pH: 7.5 Time: 15 min	Shake-flask	NA	Zhu et al. (2013)
Immobilized NitCom	-	Nitrilase	10 mM 3-cyanopyridine	Temp: 50°C, pH: 7.4 Time: 30 days	Packed bed reactor	87.5%	Teepakorn et al. (2021)
*Rhodococcus zopfii* (RzNIT/W167G)	*Escherichia coli*	Nitrilase	300 mM 2-chloronicotinonitrile	-	Batch	90%	Dai et al. (2022)
Gordonia terrae	*Gordonia terrae* mutant MN12	Nitrilase	100 mM 3-cyanopyridine	Temp: 40°C, pH: 8.0 Time: 15 min	Batch	100%	Monika et al. (2023)
*Acidovorax facilis* 72W	*Escherichia coli*	Nitrilase	0.8 M 3-cyanopyridine	Temp: 30°C, pH: 7.0 Time: 90 min	Semi-Continuous Packed-Bed Bioreactor	90%	Liu et al. (2023)

## 6 Immobilization of nitrilase for nicotinic acid production

**FIGURE 5:**
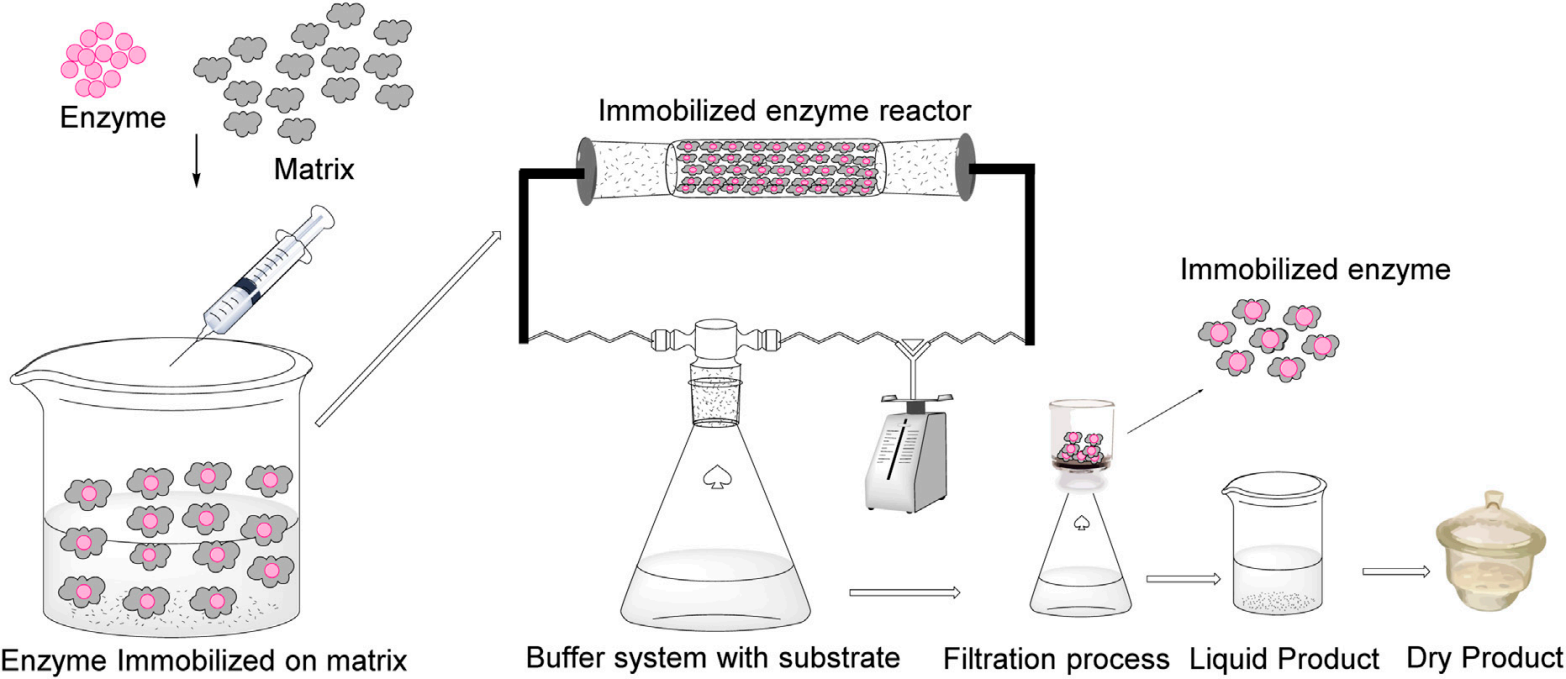
Representation of enzyme immobilization and a packed reactor.

The immobilization (Figure 5) of nitrilase has also been performed by various researchers to increase the stability and reusability of enzymes on an industrial scale (Teepakorn et al., 2021; Liu et al., 2012; Dong et al., 2017). For commercial use of nitrilase, researchers have utilized various matrices for nitrilase immobilization. The most common matrices used for nitrilase immobilization are alginate and agar. Natural polymers are often chosen because of their biocompatibility, ease of use, and ability to form stable gels under mild conditions. Recombinant *Escherichia coli* was immobilized using an alginate (2.5%) matrix, and immobilized cells were reused for up to 25 cycles with 100% activity. Finally, immobilized cells were used for a 250 mL batch reaction (1 M 3-cyanopyridine), and after 5 h of incubation, a nicotinic acid yield of 93% was obtained (Pai et al., 2014). An efficient biocatalytic process for nicotinic acid production was developed using recombinant *E. coli* JM109 cells containing nitrilase gene from *Alcaligenes* faecalis MTCC 126. The freely suspended cells demonstrated high substrate and product tolerance without inhibition. Whole-cell immobilization further enhances substrate tolerance, stability, and reusability during repeated production cycles. Under optimized conditions (37°C, 100 mM Tris buffer, pH 7.5), immobilized biocatalyst achieved 100% conversion of 1 M 3-cyanopyridine to nicotinic acid within 5 h, using 500 mg/mL fresh cell mass. The high tolerance and stability of immobilized whole-cell biocatalyst makes it a promising candidate for industrial applications in nicotinic acid production (Pai et al., 2014). A recombinant *E. coli* strain expressing nitrilase from *Acidovorax facilis* 72 W was constructed using a dual-site expression plasmid, which showed higher levels of soluble expression than the pET21a plasmid. The whole cells were immobilized using sodium alginate/glutaraldehyde/polyethylene imine, resulting in 95% activity recovery, improved stability, and 82% activity retention after 2 months of storage. A semi-continuous packed-bed bioreactor using these immobilized cells achieved efficient nicotinic acid production, with a space-time yield of 1,576 g/(L·d) at a substrate concentration of 0.8 M. The bioreactor maintained 100% conversion over 41 batches, producing 95 g of nicotinic acid at 90% yield. This technology has significant potential for industrial applications (Liu et al., 2023). Hariharan et al. (2021) investigated a nitrile-hydrolyzing enzyme from *Nocardia* globerula, NHB-2, that converts toxic nitriles into valuable amides and acids. Propionitrile at 0.3% v/v was found to be the optimal inducer. A Box–Behnken design was used to optimize the biotransformation of 3-cyanopyridine to nicotinic acid, with optimal conditions of a substrate concentration of 210 mM, a resting cell concentration of 30 U/mg DCW, and a conversion time of 70 min. Agar-immobilized cells showed improved thermal stability compared to free cells. A packed-bed reactor with immobilized cells was used for continuous nicotinic acid production, and substrate was recycled. The immobilized cells maintained 40% of their initial activity after three reuse cycles (Hariharan et al., 2021). Li et al. (2015) compared immobilized and free cells of Gibberella intermedia CA3-1 for the conversion of 3-cyanopyridine to nicotinic acid. Among the four tested samples, sodium alginate was identified as an optimal entrapment matrix. Optimal conditions for immobilization were determined to be 2% alginate, 0.6% CaCl2, 0.4 g cell/g alginate, and a 1.8 mm bead size. The immobilized cells demonstrated excellent substrate tolerance up to 700 mM 3-cyanopyridine and significantly improved thermal stability compared with free cells. They maintained efficiency for 28 batch cycles, producing 205.7 g/(g DCW) of nicotinic acid while retaining 80.55% enzyme activity. These results highlight the potential of immobilized *G. intermedia* CA3-1 cells for industrial-scale nicotinic acid production (Li et al., 2015).

Various matrices have been used for nitrilase immobilization using methods such as cross-linking and covalent bonding. Teepakorn et al. (2021) used a packed-bed reactor (40 mg of porous polymethyl-methacrylate beads) in continuous mode for production of nicotinic acid. The biotransformation reaction was performed using immobilized NitComm, NitPhym, and 3-cyanopyridine (10 mM). After 30 days of incubation, activity of immobilized NitComm decreased, and 35.1% bioconversion was achieved. In contrast, immobilized NitPhym showed complete bioconversion after 30 days of operation (Teepakorn et al., 2021). Khatik et al. (2022) conducted a study to optimize nitrilase for the hydroxylation of 2-chloroisonicotinonitrile to 2-chloroisonicotinic acid. ES-NIT-102 was identified as the best nitrilase and it was immobilized as a cross-linked enzyme aggregate (CLEA). The optimized nitrilase-CLEA showed improved stability and retained higher activity at elevated temperatures than the free nitrilases. Although substrate affinity slightly decreased, the immobilized enzyme achieved 98% conversion and 94.72 g/L product (>99% purity determined using high-performance liquid chromatography) formation in 24 h. Nitrilase-CLEAs remain active for three cycles, maintaining high conversion rates and product yields (Khatik et al., 2022). An automated instrument for the preparation of microspheres was developed to immobilize cells as efficient biocatalysts. This process optimizes the concentration of the polymer solution, crosslinking agents, and other conditions to produce small, uniform, highly porous microspheres. The conversion conditions for the transformation of 4-cyanopyridine to isonicotinic acid were optimized to reduce mass-transfer limitations and improve stability. The resulting immobilized cellular microspheres demonstrated impressive durability and efficiency, converting 4.6 mol/L of 4-cyanopyridine and producing 566 g/L of isonicotinic acid over 23 continuous batch cycles, demonstrating their potential as long-lasting and highly effective biocatalysts (Qian et al., 2021). Long et al. (2019) conducted a study to resolve the limitations of free nitrilase enzymes in nitrile biotransformation by employing a combination of immobilization of resting cells. Chitosan and polyvinyl alcohol were used for encapsulation under optimized conditions of 80 g/L polyvinyl alcohol, 40 g/L chitosan, and a saturated boric acid solution containing 60 g/L sodium tripolyphosphate. The immobilized cells showed significantly improved thermal and storage stabilities compared with free cells. In a feeding-batch reaction, immobilized cells produced 208 g/L nicotinic acid from 3-cyanopyridine over 525 min. These results provide a foundation for the practical application of nicotinic acid bioproduction, potentially reducing production costs by improving enzyme stability and reusability (Long et al., 2019). The cell-free extract of *Aspergillus niger* K10 was immobilized on a HiTrap Butyl Sepharose column. The immobilized enzyme showed stable activity at pH 8.0°C and 35°C. It efficiently converted 3-cyanopyridine and 4-cyanopyridine, while maintaining high activity for extended periods. The process produced nicotinic acid and isonicotinic acid (molar ratio ≈ 16:1) as the main products, with smaller amounts of their respective amides (isonicotinic acid and isonicotinamide, with a molar ratio ≈ 3:1) (Vejvoda et al., 2006). Malandra et al. (2009) compared nitrilases from Aspergillus niger K10 and Fusarium solani O1 for 4-cyanopyridine conversion in continuous-stirred membrane reactors. F. solani O1 nitrilase showed higher stability and selectivity at 40°C. Immobilized as CLEAs, it maintained >90% conversion for 52 h. Using two reactors in series with F. solani O1 nitrilase and Rhodococcus erythropolis A4 amidase increased the isonicotinic acid purity from 98% to >99.9% by hydrolyzing isonicotinamide by-product (Malandra et al., 2009). On citing literature, it has been observed that, previously, for nitrilase immobilization, most of the traditional matrixes like alginate beads, crosslinked enzyme aggregates (CLEAs), and chitosan matrixes dominate the field. However, alginate beads showed the most promising results for nitrilase immobilization in terms of reusability. Still, there is a significant lack of research utilizing advanced nanomaterials, such as metal nanoparticle-based supports, magnetic nanoparticles, carbon nanotubes (CNTs) and their derivatives, or metal-organic frameworks (MOFs). Therefore, the application of these novel nanomaterials for nitrilase immobilization appears to be a promising area for future research and development, potentially offering significant improvements in enzyme stability, activity, and reusability.

## 7 Conclusions and future perspectives

The global niacin and nicotinic acid market is expecting steady growth, with projections indicating an increase from USD 1.75 billion in 2023 to USD 2.36 billion by 2032, growing at a compound annual growth rate (CAGR) of 3.3%–3.5%. The pharmaceutical segment is driving significant demand owing to the cholesterol-lowering properties and cardiovascular benefits of niacin. Key market drivers include increasing consumer awareness of health benefits, increasing prevalence of skin-related diseases, increased use in pharmaceuticals and cosmetics, and expanding applications in food fortification. The coronavirus disease (COVID-19) pandemic accelerated market growth by boosting interest in health supplements. North America holds a significant market share, driven by its developed healthcare infrastructure and health-conscious consumers. However, minor side effects may slightly inhibit growth. Key players such as Lonza, DSM, and BASF are expanding their production capacities, especially in high-demand regions, such as China, to meet growing market needs (Business Research Insights, 2023 https://www.businessresearchinsights.com/market-reports/niacin-and-niacinamide-market-107169 [Accessed 15 January 2025]). The food and beverage industry has also contributed significantly to market growth, with an increasing demand for functional foods and dietary supplements that incorporate niacin and nicotinic acid. To fulfil this demand, scientists have developed a biocatalyst-mediated process for the production of nicotinic acid, and this enzyme-mediated biocatalytic process is a potential alternative method to the current chemical approach. Looking ahead, the market’s future appears promising, with several key developments on the horizon. Biocatalyst-mediated processes for nicotinic acid production have emerged as potential alternatives to traditional chemical approaches. Enzyme engineering has been employed to overcome the limitations of wild-type strains and improve substrate tolerance, stability, and conversion rates. Advanced screening techniques, such as metagenomics and proteomics, are being used to discover new, efficient biocatalysts. In addition, use of immobilized enzymes is gaining attention for industrial applications, allowing enzyme reuse and continuous processing. The integration of biocatalysis with flow chemistry and microreactors offers new possibilities for process intensification, potentially leading to improved reaction kinetics and increased nicotinic acid production.
